# The significance of De Ritis ratio in patients with radiation-recurrent prostate cancer undergoing salvage radical prostatectomy

**DOI:** 10.1080/2090598X.2020.1771947

**Published:** 2020-05-31

**Authors:** Fahad Quhal, Mohammad Abufaraj, Florian Janisch, Keiichiro Mori, Ivan Lysenko, Hadi Mostafaei, David D’Andrea, Romain Mathieu, Dmitry V. Enikeev, Harun Fajkovic, Axel Heidenreich, Shahrokh F. Shariat

**Affiliations:** a Department of Urology, Comprehensive Cancer Center, Medical University of Vienna, Vienna, Austria; b Department of Urology, King Fahad Specialist Hospital, Dammam, Saudi Arabia; c Division of Urology, Department of Special Surgery, Jordan University Hospital, the University of Jordan, Amman, Jordan; d Department of Urology, University Medical Center Hamburg-Eppendorf, Hamburg, Germany; e Department of Urology, Jikei University School of Medicine, Tokyo, Japan; f Research Center for Evidence Based Medicine, Tabriz University of Medical Sciences, Tabriz, Iran; g Department of Urology, Rennes University Hospital, Rennes, France; h Institute for Urology and Reproductive Health, I.M. Sechenov First Moscow State Medical University, Moscow, Russia; i Department of Urology, University Hospital Cologne, Cologne, Germany; j Department of Urology, Weill Cornell Medical College, New York, NY, USA; k Department of Urology, University of Texas Southwestern, Dallas, TX, USA; l Department of Urology, Second Faculty of Medicine, Charles University, Prague, Czech Republic

**Keywords:** Biochemical recurrence, prognostic, SRP, aspartate aminotransferase, alanine aminotransferase

## Abstract

**Objective:**

To evaluate the clinical prognostic value of preoperative serum De Ritis ratio (DRR; aspartate aminotransferase/alanine aminotransferase) on postoperative survival outcomes in patients with radiation-recurrent prostate cancer (PCa) who underwent salvage radical prostatectomy (SRP).

**Patients and methods:**

A retrospective review was conducted of patients with radiation-recurrent PCa who underwent SRP in five tertiary referral centres from 2007 to 2015. An increased preoperative serum DRR was defined as ≥1.35. The association between DRR and postoperative outcomes was tested. Multivariate Cox analyses were performed to identify the independent predictors of biochemical recurrence (BCR), metastases-free survival (MFS), overall survival (OS), and cancer-specific survival (CSS).

**Results:**

Overall 214 patients underwent SRP, of them 98 (45.8%) with a high serum DRR were included in the study. In a multivariate analysis high DRR was an independent predictor of BCR [hazard ratio (HR) 1.79, 95% confidence interval (CI) 1.16–2.78; *P* = 0.009]. No significant association was found between preoperative DRR and MFS (HR 1.32, 95% CI 0.53–3.30; *P* = 0.55), OS (HR 2.35, 95% CI 0.84–6.57; *P* = 0.10), and CSS (HR 3.36, 95% CI 0.65–17.35; *P* = 0.15).

**Conclusion:**

Increased preoperative serum DRR is associated with the development of BCR in patients with radiation-recurrent PCa who underwent SRP. DRR might serve as an early indicator of BCR, which may facilitate recognition of potential relapse and could translate into more intense follow-up and even salvage therapy in selected patients.

**Abbreviations:**

ADT: androgen-deprivation therapy; BCR, biochemical recurrence; BCRFS: BCR-free survival; CSS: cancer-specific survival; DRR: De Ritis ratio; HR: hazard ratio; MFS: metastasis-free survival; PCa: Prostate Cancer; OS: overall survival; PLND: pelvic lymph node dissection; (EB)RT: (external beam) radiotherapy; SRP: salvage radical prostatectomy

## Introduction

It is estimated that there will be almost 174 650 new cases of prostate cancer (PCa) and 31 620 associated deaths in the USA in 2019, ranking it as the most frequent cancer and the second leading cause of cancer death in men [1].

In the past several decades, radiotherapy (RT) has played an important role in the treatment of localised PCa. However, 20–50% of patients will eventually develop recurrence and sometimes will require additional treatment [2–4]. Salvage radical prostatectomy (SRP) is one of the options for such patients, but it has a varying response rate in the literature. In a systematic review, Chade *et al*. [5] reported a 5- and 10-year biochemical recurrence (BCR)-free survival of 47–82% and 28–53%, respectively. This wide range of responses to SRP shows us the need for biomarkers that can help identify the subset of patients who are likely to benefit from salvage local therapy (e.g. SRP) compared to those who need systemic therapy [6–8].

The De Ritis ratio (DRR) [the ratio of aspartate aminotransferase (AST)/alanine aminotransferase (ALT)] has been shown to be associated with poor outcomes with non-urological [9,10] and urological malignancies [11–13]. We hypothesised that preoperative DRR would be associated with biochemical progression in patients treated with SRP for radiation-recurrent non-metastatic PCa.

To test this, we examined the role of preoperative DRR in a large cohort of patients with radiation-recurrent PCa undergoing SRP. As to our knowledge, no study to date has investigated it in this group of patients.

## Patients and methods

### Patient selection

Five participating centres provided information for men treated with SRP at their site. All patients underwent concomitant pelvic lymph node dissection (PLND). The local Institutional Review Boards approved the present study. All institutions shared the agreements before the initiation of the study and provided the necessary clinical data. The study cohort included 214 consecutive patients with radiation-recurrent PCa treated with SRP from January 2007 to December 2015. The radiotherapy (RT) modalities included brachytherapy, external beam RT (EBRT), or between distinct RT techniques (EBRT and brachytherapy, EBRT and intensity modulated RT, or EBRT and three-dimensional conformal RT).

PCa recurrence after RT was defined as a PSA level increase of ≥2 ng/mL above the nadir according to the Radiation Therapy Oncology Group-American Society for Radiation Oncology Phoenix criteria [14].

All the patients underwent a pre-SRP biopsy to confirm radiation-recurrent PCa. None of the patients had radiographic evidence of metastatic disease before SRP. Open surgical SRP, including PLND, was performed in all patients.

The preoperative clinical and epidemiological features of the patients were analysed. Analysis of the receiver‐operating characteristic curve of DRR for BCR revealed 1.35 to be the optimal cut-off; the median was 1.33. The cut-off of 1.35 had the best Youden index value; therefore, patients with a DRR of ≥1.35 were assigned to the high-DRR group, while the remaining patients (DRR <1.35) were assigned to the low-DRR group.

All prostate specimens were examined by dedicated genitourinary pathologists at each centre. The pathological stage was assigned using the 2007 American Joint Committee on Cancer TNM staging system.

### Follow-up protocol

Follow-up examinations were performed in accordance with institutional protocols. Generally, the patients were followed-up quarterly within the first 2 years and semi-annually thereafter. BCR was defined as a total PSA value of ≥0.2 ng/mL after SRP. After SRP, no patient had received androgen-deprivation therapy (ADT) before the diagnosis of BCR. Distant metastases were identified using radiological imaging. The cause of death was determined by the treating physicians, by medical record review corroborated by the death certificate, or by the death certificates alone. In cases for which death certificates were retrieved and reviewed for the cause of death, only men with known recurrence after SRP, who had documented metastatic PCa, and who had PCa listed in the death certificate were considered to have died from PCa. The follow-up duration was calculated from the date of surgery to the date of death or the last follow-up visit.

### Statistical analysis

The normal distribution of data was analysed for each continuous variable using kurtosis and skewness. The Student’s unpaired *t*-test, Mann–Whitney *U*-test, and Pearson chi-squared test were used to compare two independent factors.

BCR-free survival (BCRFS) and metastases-free survival (MFS) curves were generated using the Kaplan–Meier method. A log-rank test was used for pairwise comparisons of survival. Two sets of multivariable Cox proportional hazards regression models were fitted. The preoperative model was limited to clinical and epidemiological preoperative features and the postoperative model included all features available for selection. Discrimination was evaluated using the Harrell’s concordance index (C-index). Statistical significance was set at *P* < 0.05. All tests were two-sided. Analyses were performed using STATA, version 16.0 (StataCorp LP, College Station, TX, USA).

## Results

### Association with clinical and pathological features

The clinical and pathological features of the 214 men with radiation-recurrent PCa treated with SRP were stratified by DRR level and are summarised in Table 1. The median (interquartile range) follow-up was 25.3 (15–28.5) months. In all, 102 (47.7%) patients had received neoadjuvant and concomitant ADT with the RT.

**Table 1.** Clinicopathological characteristics of the 214 patients treated with SRP for radiation-recurrent non-metastatic PCa according to preoperative DRR

Variable	All	Low DRR	High DRR	*P*
Patients, *n* (%)	214	116 (54.2)	98 (45.8)	
Age at SRP, years, median (IQR)	69 (64–72)	69 (64–73)	68 (64–72)	0.41
Body mass index, kg/m^2,^ median (IQR)	24 (24–27)	24 (24–27)	24 (24–27)	0.058
Gleason score before RT, *n* (%)				0.45
6	91(51.12)	48 (50.53)	43 (51.81)	
7	64 (35.96)	32 (33.68)	32 (38.55)	
≥8	23 (12.92)	15 (15.79)	8 (9.64)	
Tumour stage before SRP (*n* = 213), *n* (%)				0.74
T1	99 (46.48)	56 (48.28)	43 (44.33)	
T2	84 (39.44)	43 (37.08)	41 (42.27)	
≥T3	30 (14.08)	17 (15.66)	13 (14.40)	
Total PSA level before SRP, ng/mL, mean (range)	3.82 (2.05–6.5)	3.68 (1.8–6.27)	4.2 (2.2–7.1)	0.17
Gleason score before SRP, *n* (%)				0.61
6	48 (22.43)	29 (25)	19 (19.39)	
7	104 (48.60)	54 (46.55)	50 (51.02)	
≥8	62 (28.97)	33 (28.45)	29 (29.59)	
Pathological Gleason score, *n* (%)				0.62
6	14 (6.54)	9 (7.76)	5 (5.10)	
7	114 (53.27)	63 (54.31)	51(52.04)	
≥8	86 (40.19)	44 (37.93)	42 (42.86)	
Positive surgical margins, *n* (%)	43 (20.09)	23 (19.83)	20 (20.41)	0.92
Extracapsular extension, *n* (%)	92 (42.99)	48 (41.38)	44 (44.90)	0.60
Seminal vesicle invasion, *n* (%)	67 (31.31)	33 (28.45)	34 (34.69)	0.33
Lymph node metastasis, *n* (%)	40 (18.69)	18 (15.52)	22 (22.45)	0.19

IQR: interquartile range.

Serum DRR was high (≥1.35) in 98 (45.8%) patients and low (<1.35) in 116 (54.2%). There was no statistically significant difference between the two groups with regards to the clinicopathological features.

### Association with BCR

**Figure 1.**
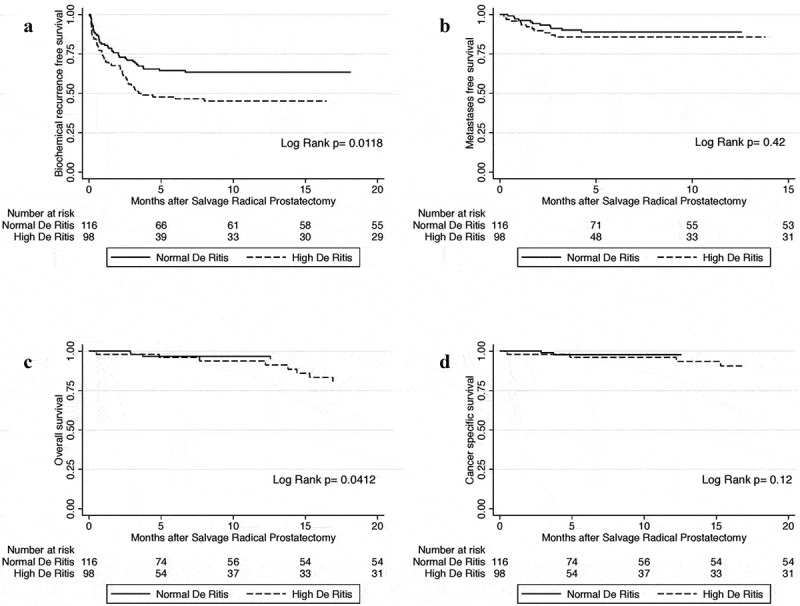
Kaplan–Meier curves showing the association of the DRR with: BCRFS (A), MFS (B), OS (C), and CSS (D); in patients treated with SRP for radiation-recurrent PCa

During follow-up, 90 (42.1%) patients developed BCR, 40 (44.4%) patients with a low DRR and 50 (55.6%) with a high DRR. Kaplan–Meier survival estimates showed a significant difference in BCRFS in patients with a low vs high serum DRR (Figure 1). On univariable analysis high DRR was significantly associated with BCR [hazard ratio (HR) 1.7, 95% CI 1.12–2.57; *P* = 0.013). On multivariable analysis, DRR retained its independent association with BCR after adjustments with established confounders in two multivariable regression models, preoperative (HR 1.79, 95% CI 1.16–2.57; *P* = 0.009) and postoperative (HR 1.7, 95% CI 1.1–2.63; *P* = 0.018). (Table 2)

**Table 2.** Pre- and postoperative multivariate Cox regression analysis of BCR

Variable	BCR			
	Univariable		Multivariable	
	HR (95% CI)	*P*	HR (95% CI)	*P*
**Preoperative**				
Total PSA before SRP	1.05 (1.03–1.07)	<0.001	1.02 (1.002–1.05)	0.031
Tumour stage before SRP				
T1	–	–	–	–
T2	1.64 (1.03–2.61)	0.039	1.31 (0.81–2.11)	0.26
≥T3	2.62 (1.33–4.46)	0.001	1.54 (0.81–2.94)	0.18
Gleason score before SRP	1.49 (1.26–1.77)	<0.001	1.39 (1.14–1.69)	0.001
DRR	1.70 (1.12–2.57)	0.013	1.79 (1.16–2.78)	0.009
Harrell’s C-index			0.6619/0.6768	
**Postoperative**				
Total PSA before SRP	1.05 (1.03–1.07)	<0.001	1.02 (0.99–1.04)	0.13
Positive surgical margins	2.55 (1.64–3.97)	<0.001	1.40 (0.84–2.36)	0.20
Extracapsular extension	2.86 (1.87–4.38)	<0.001	1.97 (1.15–3.39)	0.014
Seminal vesicle invasion	2.41 (1.59–3.67)	<0.001	1.10 (0.65–1.85)	0.72
Lymph node metastasis	3.29 (2.08–5.20)	<0.001	1.56 (0.87–2.76)	0.13
Pathological Gleason score	1.55 (1.29–1.87)	<0.001	1.32 (1.05–1.64)	0.015
DRR	1.70 (1.12–2.57)	0.013	1.70 (1.10–2.63)	0.018
Harrell’s C-index			0.7181/0.7320

Other independent pathological predictors for BCR were extracapsular extension, positive lymph nodes, and the SRP Gleason score (Table 2). Serum DRR improved the C-index by 1.5% up to 0.6768 for prediction of BCR after SRP (Table 2).

### Association with metastasis

During follow-up, 23 (10.7%) patients developed metastasis, 11 (47.8%) with a low DRR and 12 (52.2%) with a high DRR. There was no significant association between serum DRR and MFS in univariate (HR 1.40, 95% CI 0.62–3.17; *P* = 0.42) and multivariate analysis (HR 1.32, 95% CI 0.53–3.30; *P* = 0.55). Significant independent predictors of MFS were preoperative total PSA (HR 1.05, 95% CI 1.01–1.09; *P* = 0.007), biopsy Gleason score before SRP (HR 2.14, 95% CI 1.48–3.09; *P* < 0.001), positive lymph nodes (HR 3.75, 95% CI 1.35–10.43; *P* = 0.011), and pathological Gleason score (HR 2.12, 95% CI 1.37–3.28; *P* = 0.001).

### Association with OS and cancer-specific survival (CSS)

During follow-up, 18 (8.41%) patients died, seven (38.9%) patients with a low DRR and 11 (61.1%) with a high DRR. Increased preoperative serum DRR was significantly associated with OS in univariable analysis (HR 2.63, 95% CI 1.004–6.88; *P* = 0.049). However, it did not retain its statistical association in multivariable analysis when adjusted for the effects of established clinicopathological features (HR 2.35, 95% CI 0.84–6.57; *P* = 0.10).

As only seven (3.3%) patients were reported to have died from PCa during follow-up, DRR was only tested for association with CSS in univariable analysis, and showed no statistically significant association (HR 3.36, 95% CI 0.65–17.35; *P* = 0.15).

## Discussion

In the present study, we analysed 214 patients who underwent SRP for radiation-recurrent PCa. The main finding of our present study was the positive association of a high DRR with the risk of BCR in this group of patients. The significant association between a high DRR and the risk of BCR even persisted after adjustment for multiple potential confounders in the Cox-multivariate models. The association of preoperative DRR with the risk of BCR might facilitate recognition of potential relapses and could translate into more intense follow-up and even salvage therapy in selected patients. Notably, in our present study, we found an association between a high DRR and OS in univariate analysis, but not in multivariate analysis.

AST and ALT are commonly used serum biomarkers in daily clinical practice. The AST/ALT ratio is also referred to as the ‘De Ritis ratio’ from the author who first described it [15]. De Ritis first reported on the ratio as an assessment tool for disease in viral hepatitis [15], but recently it has been found to be a valuable prognostic tool for other non-hepatic diseases [16,17].

To date, several studies have reported a significant relationship between the DRR and the prognosis of various cancers, regardless of the presence of liver-specific disease [9,10,18]. Bezan *et al*. [12] retrospectively analysed 698 patients with localised RCC, and reported that the DRR could predict inferior MFS (HR 1.61, 95% CI 1.25–2.07; *P* < 0.001) and OS (HR 1.76, 95% CI 1.34–2.32; *P* < 0.001).

For PCa, a retrospective analysis of 488 patients with localised PCa by Wang *et al*. [19], found that a high DRR was an independent predictor of BCR (HR 1.72, 95% CI 1.06–2.78; *P* = 0 027). Thus, the present study aimed to evaluate the prognostic impact of the DRR in patients with radiation-recurrent PCa undergoing SRP.

The mechanisms of association of elevated AST/ALT ratio with poor outcomes of patients with cancer remain unclear. Previous studies hypothesised the involvement of AST in aerobic glycolysis, which is believed to be increased in tumour cells. AST is known to play a vital role in aerobic glycolysis by relocating cytoplasmic nicotinamide adenine dinucleotide hydrogen (NADH) into the mitochondria through malate-aspartate shuffling, which leads to the activation of more AST than ALT in rapidly growing cancer tissues [20–22].

Several limitations of the present study should be noted. First, the retrospective design and the short follow-up. Second, the AST/ALT ratio might have been biased by the presence of an undetected liver disease or a drug interaction that can affect liver function. Further studies with a prospective design are needed for validation of these results.

## Conclusion

The present study identified that preoperative serum DRR has potential for predicting BCR in patients after SRP. Patients with a high DRR were more likely to develop BCR (55.6%) than those with a lower DRR (44.4%). DRR might serve as an early indicator of BCR, which may facilitate recognition of potential relapse and could translate into more intense follow-up and even salvage therapy in selected patients.
